# A Contrast-Enhanced Computed Tomography Based Radiomics Approach for Preoperative Differentiation of Pancreatic Cystic Neoplasm Subtypes: A Feasibility Study

**DOI:** 10.3389/fonc.2020.00248

**Published:** 2020-02-28

**Authors:** Xiaoyong Shen, Fan Yang, Pengfei Yang, Modan Yang, Lei Xu, Jianyong Zhuo, Jianguo Wang, Di Lu, Zhikun Liu, Shu-sen Zheng, Tianye Niu, Xiao Xu

**Affiliations:** ^1^Department of Radiology, First Affiliated Hospital, Zhejiang University School of Medicine, Hangzhou, China; ^2^Department of Hepatobiliary and Pancreatic Surgery, First Affiliated Hospital, Zhejiang University School of Medicine, Hangzhou, China; ^3^Sir Run Run Shaw Hospital, Zhejiang University School of Medicine, Hangzhou, China; ^4^Institute of Translational Medicine, Zhejiang University, Hangzhou, China; ^5^College of Biomedical Engineering & Instrument Science, Zhejiang University, Hangzhou, China; ^6^Nuclear & Radiological Engineering and Medical Physics Programs, Woodruff School of Mechanical Engineering, Georgia Institute of Technology, Atlanta, GA, United States

**Keywords:** pancreatic cystic neoplasm, contrast-enhanced computed tomography, radiomics, differentiation diagnosis, machine learning

## Abstract

**Background:** Serous cystadenoma (SCA), mucinous cystadenoma (MCN), and intraductal papillary mucinous neoplasm (IPMN) are three subtypes of pancreatic cystic neoplasm (PCN). Due to the potential of malignant-transforming, patients with MCN and IPMN require radical surgery while patients with SCA need periodic surveillance. However, accurate pre-surgery diagnosis between SCA, MCN, and IPMN remains challenging in the clinic.

**Methods:** This study enrolled 164 patients including 76 with SCA, 40 with MCN and 48 with IPMN. Patients were randomly split into a training cohort (*n* = 115) and validation cohort (*n* = 41). We performed statistical analysis and Boruta method to screen significantly distinct clinical factors and radiomics features extracted on pre-surgery contrast-enhanced computed tomography (CECT) images among three subtypes. Three reliable machine-learning algorithms, support vector machine (SVM), random forest (RF) and artificial neural network (ANN), were utilized to construct classifiers based on important radiomics features and clinical parameters. Precision, recall, and F1-score were calculated to assess the performance of the constructed classifiers.

**Results:** Nine of 547 radiomics features and eight clinical factors showed a significant difference among SCA, MCN, and IPMN. Five radiomics features (Histogram_Entropy, Histogram_Skeweness, LLL_GLSZM_GLV, Histogram_Uniformity, HHL_Histogram_Kurtosis), and four clinical factors, including serum carbohydrate antigen 19-9, sex, age, and serum carcinoembryonic antigen, were identified important by Boruta method. The SVM classifier achieved an overall accuracy of 73.04% in training cohort and 71.43% in validation cohort, respectively. The RF classifier achieved overall accuracy of 84.35 and 79.59%, respectively. The constructed ANN model showed an overall accuracy of 77.39% in the training dataset and 71.43% in the validation dataset. All the three classifiers showed high F1 score for differentiation among the three subtypes.

**Conclusion:** Our study proved the feasibility and translational value of CECT-based radiomics classifiers for differentiation among SCA, MCN, and IPMN.

## Introduction

Pancreatic cystic neoplasm (PCN) has been estimated to be present in 2–45% of the general population (1, 2). As computed tomography (CT) and magnetic resonance imaging (MRI) become widely used in clinical work, the incidence of PCN has increased to 3–13% for individuals undergoing cross-sectional imaging (3–5). Serous cystadenomas (SCA), mucinous cystic neoplasm (MCN), and intraductal papillary mucinous neoplasm (IPMN) constitute a majority of the PCN subtypes encountered in practice (6, 7). SCA is of benign nature and periodical surveillance is enough (8). MCN, IPMN are with the degree of malignancy, and thus close surveillance and radical surgery are recommended (8–10).

The pre-surgery classification of PCN subtypes is crucial for making personalized treatment strategies. However, it is still challenging to achieve an accurate differential diagnosis (9, 11, 12) preoperatively in the clinic. Till now, no nucleic acid or protein biomarkers in blood are available to precisely differentiate PCN subtypes in clinical work. DNA markers in cyst fluid, like GNAS, show potential in identifying mucin-producing cyst lesions but far from the bench. The differentiating value of RNA or non-carcinoembryonic antigen (CEA) protein markers is still lacking sufficient evidence (10, 13). Brugge et al. claimed cyst fluid CEA level (>192 ng/mL) could differentiate mucinous from non-mucinous lesions with an accuracy of 79%, while cystic fluid carbohydrate antigen (CA 19-9) (>2,900 U/mL) presented a sensitivity of 68% and specificity of 62% (13, 14). As for radiology method, radiological examination (CT/MRI/Magnetic Resonance Cholangiopancreatography) has limited diagnostic accuracy, even by experienced radiologists. Endoscopic ultrasound (EUS)-based diagnosis methods like endoscopic ultrasound (EUS) guided fine needle aspiration (FNA) should be performed only when diagnosis of CT or MRI are unclear (10). The limit of current methods will hamper the making of proper medical decisions, increase the suffering of the patients and waste of limited medical resources. Thus, a reliable approach for classifying the subtypes of PCN per-surgery is urgently needed to facilitate personalized medicine.

Past decades had witnessed the rapid development of the field of medical image analysis, facilitating the development of the radiomics method which quantifies the tumor heterogeneity into high-dimension features (15). The radiomics approach can help clinicians make individualized decisions based on the quantitative radiomics features and machine-learning-based models (16). Chakraborty et al. investigated the CT based radiomics features as markers for stratifying the high-risk IPMN patients (17). However, the potential of radiomics methods in helping accurate diagnose of subtypes of PCN has yet been fully investigated.

Although MRI is the preferred modality according to the 2018 European evidence-based guideline (10), in developing countries like China, South America, and Africa, MRI is not always accessible. Contrast-enhanced CT (CECT) is the main diagnosis modality for PCN in China. In our center, SCA, MCN, and IPMN are most common subtypes. From retrospective analysis of pre-surgery radiological diagnoses and pathological examination results, we found diagnosis of SCA and MCN were either obscure or wrong. And IPMN was the main misdiagnosed type for both SCA and MCN. Therefore, in this study, we aimed to investigate the feasibility of using CECT based radiomics approach for preoperatively classifying SCA, MCN, and IPMN to facilitate the personalized treatment for patients with PCN.

## Materials and Methods

### Patients

Patients with pancreatic lesions treated from January 2014 to March 2019 in our center were retrospectively evaluated. Patients with pathologically proven SCA, MCN, and IPMN were selected for further analysis. The inclusion criteria were as following: (i) patients had undergone a CECT scan within 2 weeks before surgery; (ii) patients had postoperative pathological diagnosis of SCA, MCN or IPMN. The exclusion criteria were: (i) patients diagnosed with concurrent hepatic-pancreato-biliary malignancies, such as hepatocellular carcinoma; (ii) patients whose CT images were affected by strong imaging artifacts, i.e., artifacts obscuring more than 10% of whole volume of interest; (iii) patients whose clinical data or CT images were missing. Collected clinical data includes patient age, gender, abdominal symptoms (including abdominal pain, diarrhea and obscure abdominal discomfort), tumor location (head and neck, body and tail, both), calcification, tumor maximum diameter, serum platelet count, serum alanine aminotransferase (ALT), serum aspartate aminotransferase (AST), serum albumin (ALB), serum fasting blood glucose (FBG), serum tumor markers [alpha-fetoprotein (AFP), CEA, CA19-9, and serum ferritin (SF)], familial history of pancreatic cancer, chronic pancreatitis history, history of smoking, history of alcoholic consumption, obesity [based on body mass index (BMI), patients with BMI equal to or larger than 25 were identified as obesity], and blood type. The final enrolled patient dataset was randomly split into independent training group (70%) and validation group (30%), using a stratified sampling method (18). Ethical approval was obtained from Human Research Ethics Committee (HREC) of our hospital. The patient informed consent was waived by the HREC for the retrospective usage of patients' medical images.

### Study Design

**Figure 1:**
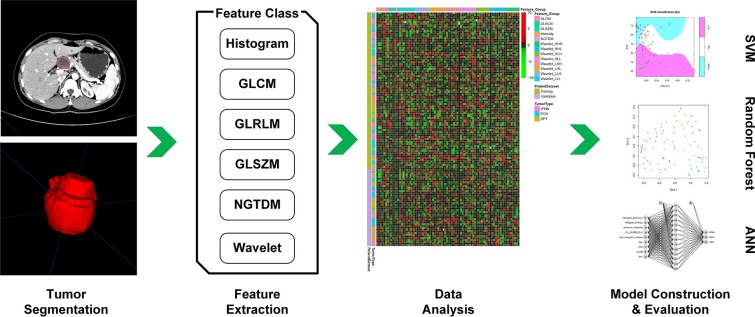
First, we performed delineation of the region of interest and segmentation, then features belong to different categories (Histogram, GLCM, GLRLM, GLSZM, NGTDM, and wavelet) were extracted and further analyzed. According to feature selection algorithm, the most important features were selected for model construction. Then, the performance of constructed model was evaluated in the validation dataset.

The analysis workflow of this study was shown in Figure 1. After delineation and segmentation of the region of interest, features belong to different categories [histogram, gray-level co-occurrence matrix (GLCM), gray-level run-length matrix (GLRLM), gray-level size zone matrix (GLSZM), neighborhood gray-tone difference matrix (NGTDM) and wavelet] were extracted and analyzed. Then the most important features were selected for model construction using supporting vector machine (SVM), random forest (RF) and artificial neural network (ANN) algorithm.

### Image Acquisition

The preoperative CECT images of patients were retrieved from the Picture Archiving and Communication Systems in our institution. All scans were performed on a 256-Slice CT scanner (Brilliance iCT, Philips, Cleveland, OH, USA) in our hospital. The scan voltage was 100 or 120 kV and the scan current was 110–835 mAs, adjustable for different patient conditions. The CECT images were reconstructed with a standard kernel. The reconstruction slice thickness was 3–5 mm and the pixel spacing of CT images ranged from 0.5 to 1 mm. The scan is performed after a 60 s delay following intravenous administration of 1.5 ml/kg of iodinated contrast medium (Iohexol Injection, 300 mg I/ml, Ousu, Yangtze River Pharmaceutical Group) and 20 ml of saline at a rate of 3 ml/s with an automatic pump injector. Arterial phase was carried out at 25–35 s after contrast injection and CT scans of arterial phase were used for subsequent process.

### Tumor Segmentation and Quantification

**Figure 2:**
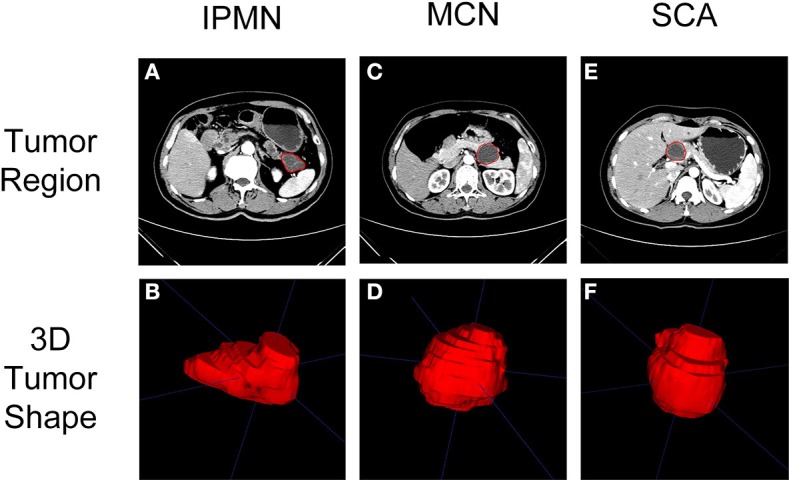
Typical CT imaging (arterial phase) of SCA, MCN, and IPMN were shown in **(A,C,E)**. The region of interest on CT imaging after delineation were shown in **(B,D,F)**.

The arterial phase of the CECT scan showed an enhanced pattern of the tumor region (19) and thus was selected for quantifying the tumor heterogeneity in this study. The delineation of tumor regions was performed, on all 3D CT slices, by a board-certified radiologist using ITK-SNAP [*www.itksnap.org* (20)]. The radiologist was blind to the clinical information before performing segmentation. The final tumor regions of patients were checked and agreed by a senior radiologist. The sample delineation results of SCA, MCN, and IPMN were shown in Figure 2. The uncertainty of tumor segmentations contributes to the variation of radiomics feature extraction which is challenging for the reproducibility of radiomics study, as reported in previous studies (21, 22). It is important to screen radiomics features that are robust against tumor segmentation uncertainty. In this study, we conducted a random expansion and corrosion process on the initial tumor region to mimic the uncertainty of manual tumor segmentation. Each slice of the initial tumor segmentation was controlled by a random seed to expand, corrode or keep unchanged. The range of expansion and erosion was 1–4 pixels, controlled by a random seed. By mimicking the tumor segmentation uncertainty, another two sets of tumor regions were generated.

The tumor region on CT images was quantified as quantitative features, namely radiomics features, for building classifier purposes. To eliminate the effect of different voxel spaces on feature extraction, the voxel size of images was resampled into a normalized, 1*1*5 mm^3^, voxel size and all the tumor regions were quantified as 64 gray levels (23) to normalize the inhomogeneity of datasets due to variable tube voltages. The histogram of the tumor region was quantified as seven features, which are variance, skewness, kurtosis, mean, energy, entropy, and uniformity. The textures of the tumor region were quantified using the GLCM, GLRLM, GLSZM, NGTDM methods. The wavelet transform was used to decompose the images into eight images of different scales to enforce the information in different directions. A total of 547 radiomics features were extracted from the tumor region in this study. The details of the feature quantification method can be found in the study of Vallieres et al. (24). The feature extraction was implanted on the MATLAB 2017b.

### Feature Selection and Classifier Construction

Three sets of radiomics features were extracted for robust feature selection, using tumor regions delineated by radiologists and generated using random expansion or corrosion. The radiomics features with an intraclass correlation coefficient of higher than 0.75 were selected for model construction (25). Further, the intercorrelation among radiomics features was assessed to exclude the highly inter-correlated radiomics features (correlation coefficient > 0.75, Pearson) from this study. Only radiomics features and clinical factors that were significantly different among three subtypes were selected.

Then the Boruta algorithm was used for further feature selection (26). Boruta algorithm uses a wrapper method based on the RF classifier for feature selection. A “shadow” attribute was created for each feature in the feature pool by shuffling values of the original feature across all patients. Then the shadow attributes are combined with original features for classification using an RF model. The importance of shadow attribute is used as a reference for selecting truly important features, as determined by RF permutation importance measure.

The multi-class classifiers using the SVM, RF, and ANN models were built based on the final selected features in the training dataset. For SVM modeling, 4 kinds of the kernel were tested, which are “Linear,” “Laplacian,” “Gaussian,”and “ANOVA RBF.” The cost of constraints violation (C-value) ranging from 1 to 10 was tested. For RF modeling, the number of variables randomly sampled as candidates at each split and total tree numbers was tested. For ANN modeling, the number of units in the hidden layer of the network and the parameter for weight decay were optimized using a grid-search strategy. The mean errors for SVM, mean out-of-bag (OOB) errors for RF and accuracy for ANN in 4-fold cross-validation were used to determine the optimal parameters for constructing the SVM, RF, and ANN models. Then the developed models were validated on the independent validation dataset.

For multi-class classification analysis, the precision, recall, and F1-score are suitable to assess the agreement between true class and predicted the result (27). As such, in this study, for characterization of three subtypes of PCNs, the precision, recall and F1 score of each subtype and overall accuracy were used to access the prediction performance of the proposed radiomics SVM and RF models. The precision is used to evaluate the accuracy for users. For example, the precision for IPMN is defined as the rate of truly predicted IPMN patients in all the patients who are predicted as IPMN. The recall is used to evaluate the accuracy of classifier, i.e., the recall for IPMN is defined as the rate of truly predicted IPMN patients in all the IPMN patients. F1 score is an indicator of comprehensively evaluating the performance of a classifier. The F1 score is defined as:

$$\begin{array}{l} \text{F}1=\frac{2\times Precision\times Recall}{\left(Precision+Recall\right)} \end{array}$$

### Statistical Analysis

The Kruskal-Wallis test was performed to evaluate the difference of the radiomics features and continuous clinical factors among three sub-types. The chi-squared test, corrected chi-square test, and Fisher test were performed to find significant different categorical clinical factors among three subtypes, where appropriate. All the statistical analyses and classifier construction were performed with R 3.4.1 (www.R-project.org, 2016). The Boruta feature selection was based on the package “Boruta” in R. The R package “kernlab,” “RandomForest,” and “nnet” were implanted in the construction of the SVM, RF and ANN model, respectively.

## Results

### Patient Characteristics

From January 2014 to March 2019, 91 patients were pathologically diagnosed with SCA. Of 91 SCA patients, 15 patients were excluded (one with concurrent malignancy, one patient was sent to our center for emergency exploratory laparotomy, 10 patients' preoperative CT images were missing, three patients' clinical data were missing). Forty-eight patients were pathologically diagnosed with MCN. Of 48 MCN patients, eight patients were excluded (two with concurrent malignancies, four patients' preoperative CT images were missing, two patients' clinical data were incomplete). When we retrospectively analyzed the radiological diagnosis of all 139 patients, the preoperative radiological diagnosis was quite unsatisfying, with only 13.4 and 10.4% were consistent with pathological diagnosis for SCA and MCN, respectively. The most common misdiagnosis for both SCA and MCN was IPMN, indicating difficulty in imaging diagnosis between these three subtypes. Therefore, we randomly enrolled 50 IPMN patients who received surgery in our center between January 2014 and March 2019 based on post-surgery pathology diagnosis, two IPMN patients were excluded for incomplete clinical data. Finally, 164 patients were enrolled (SCA, *n* = 76; MCN, *n* = 40; IPMN, *n* = 48). The patient recruitment process and inclusion/exclusion criteria were shown in Figure S1.

The training cohort included 53 SCA patients, 28 MCN patients, and 34 IPMN patients. The validation cohort included 23 SCA patients, 12 MCN patients, and 14 IPMN patients. The patient characteristics in the two cohorts were summarized in Table 1. The two datasets showed consistent distribution in all the clinical characteristics.

**Table 1:** Patient clinical factors in training and validation cohort.

**Clinical factors**	**Training**	**Validation**	***p*-value**
Tumor type			0.9914
SCA	53	23	
MCN	28	12	
IPMN	34	14	
Age *median [range]*	57 [20–79]	57 [26–79]	0.3844
Maximum Diameter *median [range]*	3.5 [0.6–14.8]	3.3 [0.5–11]	0.4936
Serum platelet *median [range]*	199 [46–443]	202 [87–397]	0.8871
Serum ALB *median [range]*	44.4 [22.9–54.9]	45 [32.9–52.8]	0.7261
Serum ALT *median [range]*	16 [5–452]	14 [6–134]	0.2255
Serum AST *median [range]*	19 [10–280]	19 [11–68]	0.4175
Serum FBG *median [range]*	5.12 [2.65–15.03]	4.85 [3.98–7.07]	0.1699
Serum AFP *median [range]*	2.3 [0.2 −2374.9]	2.3 [0.7–5.2]	0.2905
Serum CEA *median [range]*	1.9 [0.6–682.8]	1.8 [0.6–19.1]	0.3389
Serum CA 19–9 *median [range]*	9.6 [1–8170.2]	9.8 [1–128.8]	0.9799
Serum SF *median [range]*	132.4 [4.7–23290.9]	124 [3.8–1547.2]	0.7807
Sex			0.5605
Male	35	12	
Female	80	37	
Location			0.1927
Head and neck	45	21	
Body and tail	62	28	
Other	8	0	
Number of tumors			0.3821
Single	104	47	
Multiple	11	2	
Calcification			1
Without	109	46	
With	6	3	
Chronic Pancreatitis History			1
Without	114	49	
With	1	0	
Abdominal symptom			0.4125
Without	66	24	
With	49	25	
Pancreatic neoplasm family history			1
Without	115	49	
History of smoking			0.6427
Without	101	41	
With	14	8	
History of alcoholic consumption			0.4710
Without	94	43	
With	21	6	
Blood type			0.6167
A	34	14	
B	22	6	
AB	9	6	
O	50	23	
Obesity			0.5724
Without	93	37	
With	22	12	

### Feature Selection

**Figure 3:**
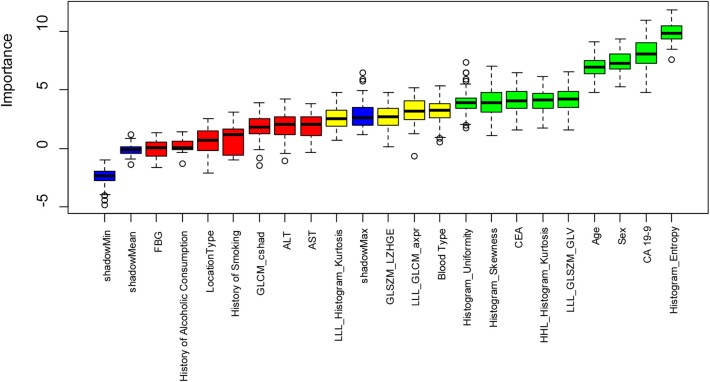
The feature importance in the Boruta feature selection process. The green box showed the features which are confirmed important, the yellow box showed the tentative attributes and the green box showed the unimportant features. Five important radiomics features include Histogram_Entropy, Histogram_ Skewness, LLL_GLSZM_GLV, Histogram_Uniformity, HHL_Histogram_Kurtosis. Four important clinical parameters include serum CA 19-9, sex, age, and serum CEA.

A total of 402 radiomics features were robust against the segmentation uncertainties. Among the robust features, 55 features with an inter-correlation coefficient of <0.75 were preliminarily selected in the training dataset. Nine radiomics features showed significant differences among the SCA, MCN, and IPMN. Nine radiomics features and eleven significant clinical factors (age, ALT, AST, FBG, CEA, CA 19-9, sex, location, blood type, cigarette history, alcoholic history) were further selected utilizing Boruta feature selection method. In the end, five radiomics features and four clinical parameters were confirmed important. The rank plot of feature importance was shown in Figure 3. The radiomics feature, Histogram_Entropy, showed the highest importance. The clinical factor, serum CA 19-9, was the second most important feature. The other 4 radiomics features were the Histogram_Skeweness, LLL_GLSZM_GLV, Histogram_Uniformity and HHL_Histogram_Kurtosis. The detailed formula of the five selected radiomics features was shown in Table S1. The other three clinical factors included sex, age, and serum CEA. The radiomics features showed comparable value with clinical factors in these selected features.

### Model Construction and Evaluation

The SVM, RF, and ANN models were constructed based on the nine important features. An SVM model with a Gaussian kernel and C-value of 2 showed the least mean error and was selected for classification of SCA, MCN, and IPMN. The detailed parameter optimization process in construction of SVM model was shown in Table S2. The constructed SVM model showed an accuracy of 73.04% in the training dataset as shown in Table 2. The precision for diagnosis of SCA, MCN, and IPMN was 74.55, 64.00, and 77.14%, respectively. In the validation dataset, the SVM model achieved an overall accuracy of 71.43%, consistent with its performance in the training cohort. The precision for each type was 68.00% for SCA, 77.78% for MCN and 73.33% for IPMN.

**Table 2:** Diagnosis performance of the constructed SVM model in the training and validation dataset.

	**Training dataset**						**Validation dataset**					
**TP**	**IPMN**	**MCN**	**SCA**	**Pre**	**Rec**	**F1**	**IPMN**	**MCN**	**SCA**	**Pre**	**Rec**	**F1**
IPMN	27	3	5	0.7714	0.7941	0.7826	11	0	4	0.7333	0.7857	0.7586
MCN	2	16	7	0.6400	0.5714	0.6038	0	7	2	0.7778	0.5833	0.6667
SCA	5	9	41	0.7455	0.7736	0.7593	3	5	17	0.6800	0.7391	0.7083
Total	34	28	53	OA	0.7304		14	12	23	OA	0.7143	

*T, True type; P, Predicted type; Pre, Precision; Rec, Recall; OA, Overall accuracy*.

**Figure 4:**
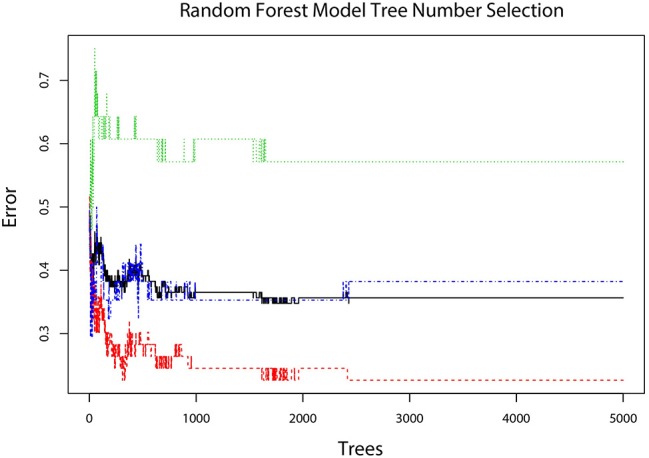
The error plot corresponding different tree numbers in the construction of the RF model. The red line showed the error of “SCA” class; the green line showed the error of “MCN” class; the blue line showed the error of “IPMN” class; the black line showed the OOB error. When tree number is more than 3,000, the errors become stable and thus 3,000 was chosen as the optimal tree number.

The error plot in selecting the tree numbers in the RF model construction was shown in Figure 4. When the tree number is more than 3,000, the errors became stable in building RF models. When two variables were randomly sampled as candidates at each split in RF, the mean OOB error was least (Table S3. Thus, the RF model with 3,000 trees and two candidate variables was established for tumor diagnosis. In the training dataset, the RF model showed 84.35% overall accuracy in the classification of SCA, MCN, and IPMN. In the validation dataset, the RF model had a precision of 72.41% for SCA, 90.00% for MCN, 90.00% for IPMN (Table 3).

**Table 3:** Diagnosis performance of the constructed RF model in the training and validation dataset.

	**Training dataset**						**Validation dataset**					
**TP**	**IPMN**	**MCN**	**SCA**	**Pre**	**Rec**	**F1**	**IPMN**	**MCN**	**SCA**	**Pre**	**Rec**	**F1**
IPMN	30	4	1	0.8571	0.8824	0.8696	9	0	1	0.9000	0.6429	0.7500
MCN	1	18	3	0.8182	0.6429	0.7200	0	9	1	0.9000	0.7500	0.8182
SCA	3	6	49	0.8448	0.9245	0.8829	5	3	21	0.7241	0.9130	0.8077
Total	34	28	53	OA	0.8435		14	12	23	OA	0.7959	

*T, True type; P, Predicted type; Pre, Precision; Rec, Recall; OA, Overall accuracy*.

**Figure 5:**
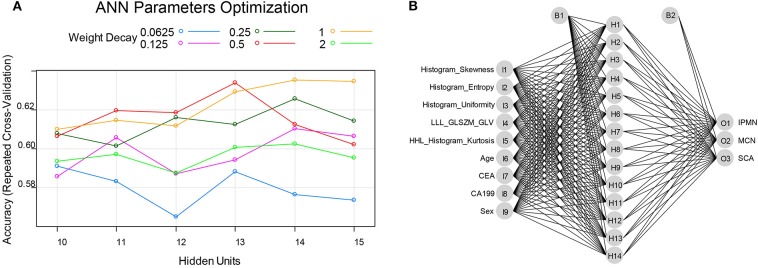
**(A)** the ANN parameters optimization process: when the hidden units were 14 and the weighted decay was 1, the accuracy reached highest and thus the ANN model was constructed with 14 hidden units and the weighted decay value of 1. **(B)** the final constructed ANN model in this study.

The number of hidden units was selected from 10 to 15 and the weight decay was chosen from 2, 1, 0.5, 0.25, 0.125, and 0.0625 in the cross-validation process of ANN structure optimization. The accuracy of ANN in optimizing the number of hidden units and weight decay was shown in Figure 5A. When the hidden units are 14 and the weight decay is 1, the mean accuracy in the cross-validation reached the highest and the corresponding ANN structure was shown in Figure 5B. The constructed ANN showed an overall accuracy of 77.39% in the training dataset and 71.43% in the validation dataset (Table 4). The precision of SCA in the validation dataset was 77.78%. For MCN and IPMN, the precisions were 66.67 and 68.42%, respectively.

**Table 4:** Diagnosis performance of the constructed ANN model in the training and validation dataset.

	**Training dataset**						**Validation dataset**					
**TP**	**IPMN**	**MCN**	**SCA**	**Pre**	**Rec**	**F1**	**IPMN**	**MCN**	**SCA**	**Pre**	**Rec**	**F1**
IPMN	31	4	7	0.7381	0.9118	0.8158	13	1	5	0.6842	0.9286	0.7879
MCN	1	15	3	0.7895	0.5357	0.6383	0	8	4	0.6667	0.6667	0.6667
SCA	2	9	43	0.7963	0.8113	0.8037	1	3	14	0.7778	0.6087	0.6829
Total	34	28	53	OA	0.7739		14	12	23	OA	0.7143	

*T, True type; P, Predicted type; Pre, Precision; Rec, Recall; OA: Overall accuracy*.

The RF model showed the highest overall accuracy in both the training and validation dataset, showing the advantage of RF models in the differential diagnosis of SCA, MCN, and IPMN. As for F1-score, the RF model showed higher F1-score for SCA and MCN, but lower F1-score for IPMN than SVM and ANN model. ANN model showed the highest F1-score for IPMN in the validation dataset. The performance of the three developed models in this study demonstrated the feasibility of models constructed with radiomics and clinical features in the diagnosis of SCA, MCN, and IPMN.

## Discussion

In this study, we investigated the potential of the radiomics method for classification of three subtypes of pancreatic cystic neoplasm, i.e., SCA, MCN, and IPMN. All the radiomics features used in the final models developed in this study were robust against tumor segmentation uncertainty. Five radiomics features and four clinical factors were identified important and used for classifier construction.

Three reliable machine learning methods, SVM, RF and ANN methods, were utilized to construct diagnostic classifiers. The built SVM model showed an overall accuracy of 73.04% for training and 71.43% for validation. The RF model showed an overall accuracy of 84.35 and 79.59% in two independent datasets. As for ANN, the overall accuracy in two independent datasets was 77.39 and 71.43%, respectively. All three classifiers present good performance in distinguishing SCA from MCN and IPMN. The result showed that the CECT based radiomics method could classify three subtypes of PCN and may help make personalized treatment decisions preoperatively.

Now the clinical management of patients with pancreatic cystic neoplasm is mainly based on clinical presentation and radiological examinations. EUS-based methods are not routinely performed in every medical center. From the retrospective comparison between preoperative radiology diagnosis and postoperative pathology diagnosis in our center, the pre-surgery accurate diagnosis rate is very low (13.4% for SCA and 10.4% for MCN). Even in Massachusetts General Hospitals, a world-class medical center, over 20% of the cyst lesions resected for concerns about their malignant potential were entirely benign based on histopathologic examination (28). This clinical dilemma reflects the urgent need for an effective and efficient differential method of PCN.

Pancreatic cystic neoplasm is heterogeneous, while the radiologists' diagnosis or cyst fluid examination just reflects a relatively small part of the whole tumor. In this study, the classifiers were constructed by combining radiomics features with clinical factors (serum CA 19-9, sex, age, serum CEA) and showed promising differential performance. The result was consistent with previous studies. Giuseppe et al. found that age was one of the significant predictors of SCA growth (29). Leung KK et.al found elevated cystic CEA was associated with potentially malignant/malignant cysts (30). Also, Bassi et al. found that positive CEA and/or co-presence of more than two positive serum markers (CEA, CA 19-9, or CA 125) were indicative of presence of mucinous cystic tumors, i.e., MCN and IPMN (31). Our results proved that clinical factors like serum tumor markers together with radiomics features could help differential diagnosis among SCA, MCN, and IPMN.

Treatment choices are sharply different for SCA, MCN, and IPMN. As SCA is a benign entity, periodic surveillance is recommended. MCN had the potential to progress to malignancy. According to current guidelines (10), patients with MCN larger than 4 cm or symptoms should undergo surgery. Ideally, IPMNs with high-grade dysplasia or with invasive adenocarcinoma should undergo resection. But it is still difficult to differentiate low-grade dysplasia in clinical work. Over 20% of the cysts were entirely benign based on histopathologic examination and over 75% of resected IPMNs could have been safely observed (32). With the radiomics approach developed in this study for differentiating SCA, MCN, and IPMN, we might avoid the 20% wrong clinical decision.

There are some limitations to our study. Firstly, as a retrospective study based on single-center data, the sample size of each subtype is relatively small. We take some measures to avoid bias. The training and validation datasets were randomly split (ratio = 7:3) to test the robustness of the results. Multifold cross-validation was carried out in constructing the machine learning classifiers to avoid the over-fitting. However, the bias may still exist due to small sample size. Secondly, there is inevitable subjectivity in the process of manual tumor segmentation. To minimize this bias caused by segmentation uncertainty, all segmentation results were checked and approved by a senior radiologist to ensure the segmentation accuracy. The random expansion and corrosion was also performed to select robust radiomics features. To further improve the performance of CECT based radiomics method, a multicenter-based prospective study with a large study population is needed.

## Conclusions

In conclusion, our study provided preliminary evidence that CECT-based radiomics analysis was feasible and reliable to differentiate SCA, MCN, and IPMN, which is convenient, non-invasive, and repeatable. On the basis of multicenter validation, the present findings may be applicable to clinical routine.

## Data Availability Statement

All datasets generated for this study are included in the article/Supplementary Material.

## Ethics Statement

Consent for publication of patients' clinical information (including clinical symptoms, biochemistry examination, and radiology imaging) was obtained from the Human Research Ethics Committee (HREC) of First Affiliated Hospital of Zhejiang University School of Medicine. The written informed consent was obtained from the patient (or in the case of children, their parent or legal guardian).

## Author Contributions

XS, TN, and XX conceived the project. FY and PY analyzed the data and wrote the paper. MY, JZ, JW, DL, and ZL collected the data. All authors edited the manuscript.

### Conflict of Interest

The authors declare that the research was conducted in the absence of any commercial or financial relationships that could be construed as a potential conflict of interest.
