# Supplementary material for: A Contrast-Enhanced Computed Tomography Based Radiomics Approach for Preoperative Differentiation of Pancreatic Cystic Neoplasm Subtypes: A Feasibility Study
Source: Front Oncol. 2020 Feb 28;10:248. doi: 10.3389/fonc.2020.00248 (PMC7058789; doi:10.3389/fonc.2020.00248)

**Supplementary**

**Table S1**. Detailed information of the selected radiomics features in this study.

| **Feature Name** | **Formula** | **Explanation** |
| --- | --- | --- |
| Histogram_Entropy | $\sum_{i}^{N_{p}} {I(i)}^{2}$ | The Entropy of the Histogram of tumor regions. |
| Histogram_Kurtosis | $\frac{\frac{1}{N_{p}}\sum_{i}^{N_{p}} {(I(i)-\bar{I})}^{4}}{{\sqrt{\frac{1}{N_{p}}\sum_{i}^{N_{p}} {(I(i)-\bar{I})}^{2}}}^{2}}$ | The Uniformity of the Histogram of tumor regions. |
| LLL_GLSZM_GLV | $\sqrt{\frac{1}{N\times S}\sum_{i}^{S} \sum_{i}^{N} {(n\times M\left( n,s \right)-\mu_{N})}^{2}}$  $\mu_{N}=\frac{1}{N\times S}\sum_{i=1}^{N} \sum_{j=1}^{S} n\times M\left( n,s \right)$ | The Gray Level Variance of Grey-Level Size-Zone Matrix of tumor regions transformed by performing low-pass wavelet filter on all 3 axes. |
| Histogram_Uniformity | $\sum_{i}^{N} {H(i)}^{2}$ | The Uniformity of the Histogram of tumor regions. |
| HHL_Histogram_Kurtosis | $\frac{\frac{1}{N_{p}}\sum_{i}^{N_{p}} {(I(i)-\bar{I})}^{4}}{{\sqrt{\frac{1}{N_{p}}\sum_{i}^{N_{p}} {(I(i)-\bar{I})}^{2}}}^{2}}$ | The Kurtosis of Histogram of image transformed by performing high-pass wavelet filter on x- and y- axis and low-pass wavelet filter on z-axis. |

**Notation:**

$N_{p}$ is the number of voxels in the tumor region.

$I(i)$ is the gray level of the i-th voxel in the tumor region.

$H(i)$ is the number of voxels with gray-level i in the histogram of image.

$M(n,s)$ is the (n, s) th value of Gray-Level Size-Zone Matrix

$N$ is the number of gray-level bins.

$S$ is the number of zone-size bins.

**Table S2**. Parameter Optimization in construction of SVM model;

| **Kernel**  **C-value** | **Linear** | **Laplacian** | **Gaussian** | **ANOVA RBF** |
| --- | --- | --- | --- | --- |
| 1 | 0.4954 | 0.4433 | 0.4954 | 0.4341 |
| 2 | 0.4692 | 0.4353 | **0.4166*** | 0.5052 |
| 3 | 0.4861 | 0.5046 | 0.4711 | 0.4255 |
| 4 | 0.4433 | 0.4784 | 0.5034 | 0.4708 |
| 5 | 0.4874 | 0.4430 | 0.4535 | 0.4433 |
| 6 | 0.5123 | 0.4172 | 0.4689 | 0.4888 |
| 7 | 0.4865 | 0.4781 | 0.4433 | 0.4452 |
| 8 | 0.4621 | 0.4347 | 0.4701 | 0.4437 |
| 9 | 0.5311 | 0.4267 | 0.4778 | 0.5043 |
| 10 | 0.4957 | 0.4440 | 0.4430 | 0.4360 |

**Table S3.** Parameter optimization in construction of Random Forest Model;

| **Mtry** | 1 | 2 | 3 | 4 | 5 | 6 | 7 | 8 | 9 |
| --- | --- | --- | --- | --- | --- | --- | --- | --- | --- |
| Mean OOB Error | 0.3747 | **0.3630*** | 0.3671 | 0.3889 | 0.3840 | 0.3830 | 0.3885 | 0.3951 | 0.3898 |

Mtry: number of variables randomly sampled as candidates at each split; mean OOB error: out-of-bag error

***Figure S1* study population enrollment**The whole process of study population enrollment. 189 patients from January 2014 to March 2019 were eligible at first. After further examination on clinical parameters and CT imaging, 25 patients were excluded. Finally, 164 patients were enrolled for further analysis.


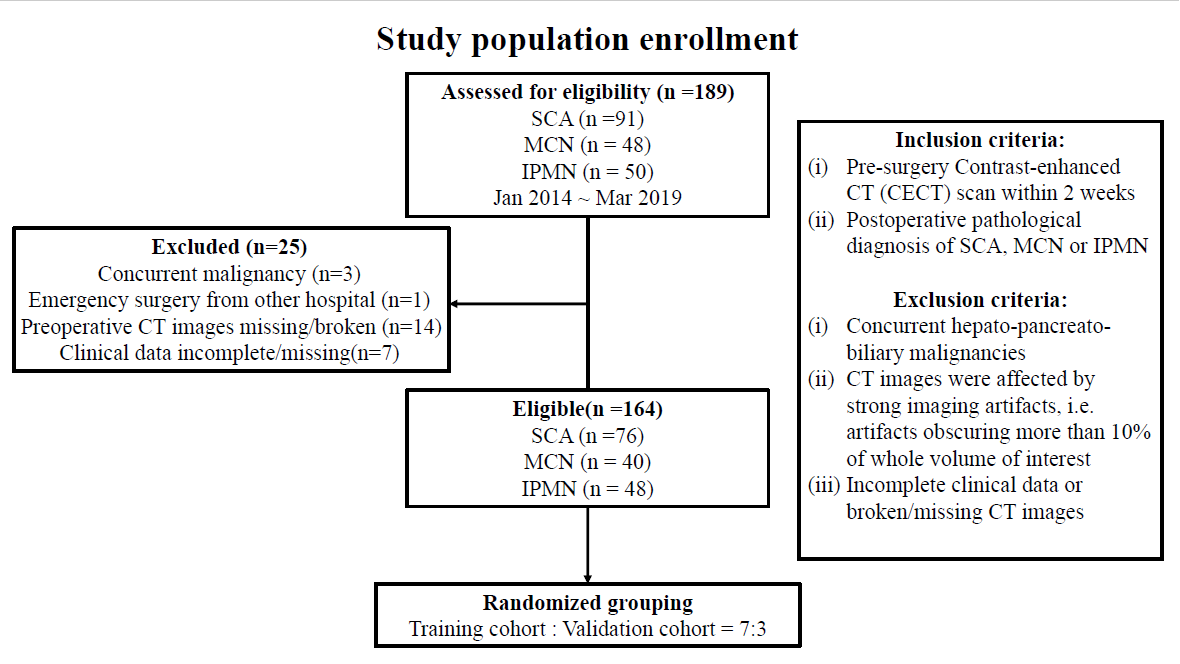

Supplement: Supplementary file 1 [file Data_Sheet_1.docx]
